# Multiple cutaneous infections caused by *Prototheca wickerhamii*

**DOI:** 10.1002/jcla.23492

**Published:** 2020-07-21

**Authors:** Feng Zhao, Miaode Chen, Ying Fu

**Affiliations:** ^1^ Department of Clinical Laboratory Sir Run Run Shaw Hospital School of Medicine Zhejiang University Hangzhou China; ^2^ Department of Emergency Surgery The First People's Hospital of Wenling Wenling China

**Keywords:** cutaneous infection, identification, *Prototheca wickerhamii*, susceptibility, treatment

## Abstract

**Background:**

*Prototheca* species are a group of organisms ubiquitously existing in nature but have become a pathogenic threat to public health, which has aroused wide attention. Species identification and antifungal susceptibility have essential and valuable meanings to clinical diagnosis and treatment.

**Methods:**

A case of an 84‐year‐old patient who had suffered from multiple cutaneous infections was reported. Tissue samples of the damaged skin were collected from the patient and used for microscopic examination and tissue culture. Staining methods, the VITEK system with YSD card and the molecular identification method based on partial mitochondrion‐encoded cytochrome b (*cytb*) gene amplification and sequencing were used for species identification. Antifungal susceptibility testing was completed by using YeastOne plate.

**Results:**

The patient had type II diabetes mellitus. Round, grape‐like, and scattered morula forms were observed under the microscope in bright blue with lactophenol cotton blue staining and in green fluorescence with fungus fluorescence staining. Yeast‐like colonies were grown on both the blood plates and the Sabouraud agar. *P wichehamii* was identified and presented resistance to three echinocandins, fluconazole, and 5‐fluorocytosine, while was susceptible to amphotericin B, posaconazole, itraconazole, and voriconazole.

**Conclusion:**

Our result revealed that an old patient with diabetes mellitus might be a dangerous population of cutaneous protothecosis. It also highlighted the contribution to microbial methodology on the diagnosis and treatment of such rare fungus infection.

## 1 INTRODUCTION

Human protothecosis is a microalgae infection caused by members of genus *Prototheca*, which was rare but has become a threat with an increasing tendency, especially in recent years.^1^ It was more commonly reported in patients who were under the immunosuppressed condition, and was easily misdiagnosed.^2^ Here, we report a case of cutaneous protothecosis in an old patient who had type II diabetes mellitus without clinical signs of the compromised immune system, and share our experiences provided by microbiology laboratory on the diagnosis and treatment of protothecosis.

## 2 CASE REPORT

### 2.1 Materials and methods

#### 2.1.1 Medical history of the patient and clinical data

On August 23, 2018, an 84‐year‐old man, who had suffered from skin ulcer and soft‐tissue necrosis at multiple sites of limbs for three months, was admitted to the First People's Hospital of Wenling. Three months ago, he had skin edema under no apparent predisposing cause. The edema was initially single and limited at his right ankle alone, while was gradually developed to be multiple, and then was aggravated to necrosis ulcers within 2 weeks. He was diagnosed as a soft‐tissue infection (bacteria being considered firstly) at Sir Run Run Shaw Hospital, and received both surgical debridement and irrigation, and antibiotics treatment (Rocephin®, ceftriaxone sodium, 2 g, qd) for 3 days. He did not test any laboratory examinations and went back home after treatment. However, the skin lesions did not recover but disseminated to the right arm at the follow‐up 2 months. To get further treatment, he visited the First People's Hospital of Wenling and was admitted to the Department of Burns with the impression of soft‐tissue infection (unknown reason).

**FIGURE 1:**
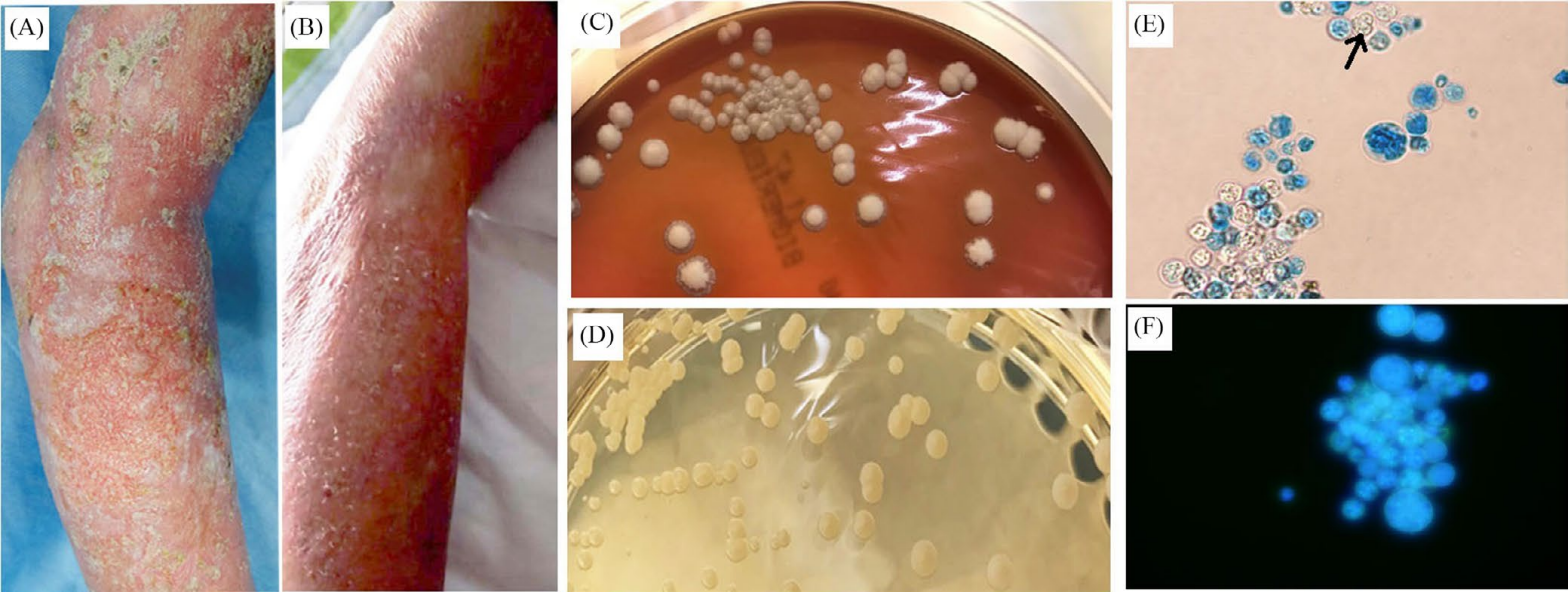
Clinical characteristics of cutaneous *protochecosis*, and morphological and microscopic feature of *Prototheca wickerhamii*. A, Skin of the right arm after debriding with 0.9% sodium chloride before treatment shows cutaneous necrosis or necrotic ulcer. B, Skin of the right arm after treatment displays the lesions have recovered. C and D, Individual colonies of *P wickerhamii* growing on Columbia agar with 5% defibrinated sheep blood and Sabouraud agar after 48 h of incubation, which are medium‐sized, round or corrugated margin, smooth or rough in colony morphology

The patient's body temperature was normal (37℃), and blood pressure was 130/70 mm Hg at the admission day. Blood laboratory tests, including white blood count (6.33 + E9/L, neutrophil percentage 73.1%), and glucose level (5.3‐5.6 mmol/L), were not abnormal except for C‐reactive protein (CRP, 103.14 mg/L). Body examination showed cutaneous necrosis, and necrotic ulcers were presenting at both the right ankle and the right arm with the largest ulcer size over 6 × 10 × 0.1 cm. The damaged skins were red with high temperature. Thickening and subcutaneous soft‐tissue edema expanded to the whole right leg and arm (Figure 1A). No other positive physical signs were observed. The patient had had comorbidities of type II diabetes mellitus for 4 years and gout for 6 years. He had received insulin therapy subcutaneously for four years. He is a farmer who is engaged in agriculture.

#### 2.1.2 Sample detection, species identification, and antifungal susceptibility test

Tissue samples were collected from the right arm's damaged skin after debriding with 0.9% sodium chloride and sent to the clinical microbiology laboratory. It was used for both direct microscopic examination and further microbial culture after being digested with 3% sodium hydroxide.

Staining methods of lactophenol cotton blue (Baso) and fungus fluorescence (Jiangsu Lifetime Biological Technology) were completed. Two plates of Columbia agar with 5% defibrinated sheep blood and Sabouraud agar were used for further culture under 37℃ and 5% CO_2_ condition_._ To identify the species of the colonies growing on the plate, the VITEK system (bioMérieux) based on biochemical reaction method with YSD card was utilized according to the manufacturers' instructions. Molecular method based on partial mitochondrion‐encoded cytochrome *b* (*cytb*) gene sequencing was involved for further species confirmation, as previously reported.^3^ To compete with the *cytb* gene sequencing, genomic DNA was first extracted using ZR Fungal/Bacterial DNA MiniPrep Kit (ZYMO research) and then PCR was performed to amplify the cytb gene following‐up Sanger sequencing at Biosum company. The sequence was then submitted to NCBI database (https://blast.ncbi.nlm.nih.gov/Blast.cgi) for alignment.

In vitro antifungal susceptibility testing was detected using YeastOne plate (Thermo Fisher Scientific) according to standard detecting procedure.

### 2.2 RESULT

#### 2.2.1 Bacterial morphology under microscope

Round, grape‐like, and scattered morula forms were observed under the microscope in bright blue and green fluorescence dying with lactophenol cotton blue and fungus fluorescence. Moruloid sporangia were composed of two to eight small sporangiospores and had a thick and highly refractile wall (Figure 1E,F).

#### 2.2.2 Culture result and species identification

After 48 hours of incubation, yeast‐like colonies were grown on both plates, which were white, mid‐sized, rough, round or corrugated margin, undulate and rough in morphology (Figure 1C,D). Both the VITEK system and the sequence of *cytb* gene showed *P wichehamii* with 100% similarity.

#### 2.2.3 Antifungal susceptibility test, clinical treatment, and outcome of the patient

*P wichehamii* showed resistance to three echinocandins (anidulafungin, micafungin, and caspofungin), fluconazole, and 5‐fluorocytosine, while was susceptible to amphotericin B, posaconazole, itraconazole, and voriconazole (Table 1).

**TABLE 1:** Minimal inhibitory concentration (MIC) of antifungal agents in *Prototheca wickerhamii*

Antifungal agent	MIC (mg/L)
Amphotericin B	1
Anidulafungin	≥8
Micafungin	≥8
Caspofungin	≥8
Posaconazole	0.5
Itraconazole	1
Fluconazole	≥256
Voriconazole	1
5‐ Fluorocytosine	≥64

The clinical diagnosis was then revised to tissue‐skin infection (human protothecosis) when the identification of *P wichehamii* was confirmed. The patient was advised to immediately treat with antifungal drugs, including fluconazole (200 mg, iv, q 12 h) and compound econazole nitrate cream on the damaged skin after debridement and irrigation (0.5 g, bid). Besides, regular insulin kept on using to control the level of blood glucose. Skin ulcers recovered within two weeks of therapy, and no more skin damage appeared. (Figure 1B) The patient was fully recovered and discharged one month later when C‐reactive protein was decreased to less than 0.8 mg/L.

## 3 DISCUSSION

Although *Prototheca* spp. have been found with low virulence, they are capable of invading the colonization skin to the mucosal cells and causing skin‐tissue infection.^2^ Here, we report a case of cutaneous protothecosis caused by *P wickerhamii* with unique and limited infectious signs on the skin tissue. We share our experience on species identification and antifungal susceptibility phenotype and emphasize the contribution of microbiology laboratory in the aspect of disease diagnosis and therapy.

Since Davies first described the infection of *Prototheca* species in 1964, human protothecosis has spread worldwide, especially in recent years.^4^, ^5^, ^6^, ^7^ In our country, cutaneous protothecosis has been reported successively in mainland China, Hong Kong, and Taiwan.^8^, ^9^ Until now, at least two protothecosis cases occurred in Shanghai, China.^10^, ^11^ Our patient is from Zhejiang Province which is close to Shanghai geographically, indicating the potential endemic area of pathogenic *Prototheca* species in the natural environment.

The traditional attitude to the *Prototheca* species is that they are of low virulence to human beings who have intact immune systems but are threatening to individuals with immunocompetent or immunosuppressed problems.^5^ Our report and some other literature hinted that old patients with diabetes mellitus were a group of vulnerable populations and should raise attention, especially when they have exposure risk to the contaminated region.^10^, ^12^

So far, the genus *Prototheca* is composed of at least eight species, like *P wickerhamii, P zopfii, P blaschkeae, P cutis, P miyajii, P stagnora, P ulmea,* and *P tumulicola*, all of which present close relationships in the biological phenotype and the genomic background.^3^ Correct species identification of *Prototheca* species would bring considerable advantages to the patient, while microbiological methods are limited because our recognition of this genus is still ambiguous.^3^, ^13^ In the microbiology laboratory, morphological methods, including microscopic morphology or colonial morphology, are useful in gross identification. Further techniques based on biochemical reactions (the VITEK) or protein profiles (the MALDI‐TOF MS) also show some capabilities. Still, sometimes the results are poorly reproducible in intraspecies identification of the genus *Prototheca*.^5^ Compared to traditional phenotype methods, molecular methods have more explicit identification schemes.^13^, ^14^, ^15^ The partial sequence of the *cytb* gene advanced by Tomasz Jagielski allows the identification of all of the currently accepted *Prototheca* species. It becomes a new genetic marker for the differentiation of *Prototheca*.^3^ Here, we applied the above methods in identifying the species of *Protocheca* and revealed *P wickerhamii* was the pathogen leading to the cutaneous infection.

In vitro susceptibility result has revealed that human *Prototheca* spp. present to be normally susceptible to amphotericin B and be variable in susceptibility to azoles, such as fluconazole, itraconazole, and voriconazole.^2^ An additional report illustrated that *P wickerhamii* was always resistant to 5‐flucytosine. Our result and Jason's research have confirmed that *P wickerhamii* isolates showed similar susceptibility profiles, which were multi‐drug resistant.^16^ So, the clinical treatment of fluconazole should be ineffective theoretically. Interestingly, in our experience, we observed that the patient's skin lesions recovered as long as treatment replaced by fluconazole and econazole nitrate cream. Our patient had the intact immunity function, and his cutaneous protothecosis was limited and mild. Therefore, we assumed that the outcome might be not only associated with the antifungal therapy but also more relevant to the adequate debridement and irrigation, and might be away from the contaminated region of *Prototheca* spp.^2^

Here, we report a cutaneous infection case of human protothecosis and emphasize the characteristics of clinical symptoms. Our result brings valuable meaning to species identification in the laboratory and treatment of protothecosis in clinical practice.

## CONFLICT OF INTEREST

The author declares that there is no conflict of interest that could be perceived as prejudicing the impartiality of the research reported.

## ETHICAL APPROVAL

The *P wickerhamii* strain was isolated from the clinical sample. This study was conducted following the Declaration of Helsinki and approved by the Ethics Committee of both Sir Run Run Shaw Hospital, School of Medicine, Zhejiang University, China, and the First People's Hospital of Wenling. The written informed consent was obtained from the patient for publication of this case report and any accompanying images.
